# Analysis of frequency-domain heart rate variability using absolute versus normalized values: implications and practical concerns

**DOI:** 10.3389/fphys.2024.1470684

**Published:** 2024-09-13

**Authors:** Youssra Amekran, Narjisse Damoun, Abdelkader Jalil El Hangouche

**Affiliations:** Department of Physiology, Faculty of Medicine and Pharmacy of Tangier, Abdelmalek Essaadi University, Tanger, Morocco

**Keywords:** autonomic nervous system, heart rate variability, frequency-domain, normalization, low frequency, high frequency

## Introduction

Heart rate variability (HRV) is a widely studied physiological phenomenon that mirrors the interplay between the sympathetic and parasympathetic divisions of the autonomic nervous system (ANS) activity (Ernst, 2017). HRV is defined as the fluctuation between consecutive heartbeats and is assessed by measuring the interval between the R-waves of the QRS complex on an electrocardiogram (ECG) (Zeid et al., 2023).

HRV quantification can be achieved using methods that are categorized as time-domain, frequency-domain or spectral density power analysis, and nonlinear methods. Time-domain parameters are the simplest to calculate and they are reflective of the variability in the R-R interval over time. The frequency-domain of HRV is estimated using spectrum analysis of the ECG signal (Zeid et al., 2023). It delineates the total variance (also known as power) of a continuous series of beats into distinct frequency components (Billman, 2013). Nonlinear methods are developed based on the need to measure the nonlinear dynamic state of the heart, and they assess the overall complexity and unpredictability inherent in HRV (Zeid et al., 2023). The different methods of HRV measurement aim to calculate numerous metrics, known as HRV parameters, quantifying the amount of variability in heart beats (R-R intervals), and are reflective of either sympathetic, parasympathetic or overall activity of the ANS activity (Heart rate variability: standards of measurement, physiological interpretation and clinical use. Task Force of the European Society of Cardiology and the North American Society of Pacing and Electrophysiology (1996)). Although the calculation of these metrics is simple, the interpretability still requires careful attention. Frequency-domain HRV metrics are widely used in the literature to measure the sympathetic and parasympathetic functions; however, some concerns related to their use need to be highlighted.

## Frequency-domain HRV: absolute versus normalized values

The frequency-domain indices calculate the amount of signal energy within component bands. The Task Force of the European Society of Cardiology and the North American Society of Pacing and Electrophysiology (1996) divided heart rate (HR) oscillations into ultra-low-frequency (ULF: ≤ 0.003 Hz), very-low-frequency (VLF: 0.0033–0.04 Hz), low-frequency (LF: 0.04–0.15 Hz), and high-frequency (HF: 0.15–0.4 Hz) band (Heart rate variability: standards of measurement, physiological interpretation and clinical use. Task Force of the European Society of Cardiology and the North American Society of Pacing and Electrophysiology (1996)). The HF and LF components are the most commonly used indices. The former is supposed to reflect the parasympathetic activity of the ANS, and the latter is, for some researchers, reflective of the sympathetic activity, but is most frequently interpreted as an indicator of the overall ANS activity (Sacha and Pluta, 2005).

These metrics can be expressed in various units: milliseconds squared (ms^2^), beats per min squared (bpm^2^), or normalized units (nu) (Heart rate variability: standards of measurement, physiological interpretation and clinical use. Task Force of the European Society of Cardiology and the North American Society of Pacing and Electrophysiology (1996); Aubert and Ramaekers (1999)). The ms^2^ unit indicates that the spectrum is derived from an R-R interval sequence, whereas bpm^2^ signifies that the spectrum is computed from an instantaneous heart rates sequence (IHRs), which are obtained by inverting the R-R interval sequence. The normalized units represent the LF and HF as a percentage of the total power (TP), typically defined as the sum of the LF and HF. These normalized values can be based on spectra expressed as either ms^2^ or bpm^2^ (Heart rate variability: standards of measurement, physiological interpretation and clinical use. Task Force of the European Society of Cardiology and the North American Society of Pacing and Electrophysiology (1996); Sacha and Pluta (2005)).

One might wonder about the appeal of the normalization process. A key part of this appeal lies in the fact that normalized spectral HRV measures are represented on a more intuitive scale, such as a proportion (0–1) or percentage (0%–100%). Moreover, normalization significantly reduces the variability within and between subjects in the raw HRV spectral power, which typically follows a long-tailed, right-skewed exponential distribution. By restricting the range of normalized values, the statistical averages of these normalized spectral indices, both within and across subjects, tend to approximate normal distributions more than do the raw spectral band power measurements (Burr, 2007). Additionally, normalization enhances the comparability of spectral HRV values across different laboratories, studies results, and algorithms of spectral density power analysis. Discrepancies in the computed band power that are related to practical concerns such as spectral analysis block length, windowing and algorithms used are generally mitigated by normalization process (Burr, 2007).

Several researchers have recommended the use of normalized units (Malliani et al., 1991; Montano et al., 2009; Reyes del Paso et al., 2013). This recommendation stems from observations showing that conditions linked to sympathetic activation result in a reduction in overall HRV power, including the LF component, while vagal activation causes the opposite effect. Hence, when spectral components are measured in absolute units, variations in the total spectral power can distort the assessment of LF and HF powers. This distortion can be avoided by using the LF/HF ratio or normalized units, which is particularly useful when examining sympathetic cardiac tone (Reyes del Paso et al., 2013). Some earlier research has suggested that when LF is presented as a normalized value, it is interpreted as a measure of pure sympathetic function (Furlan et al., 2000; Thomas et al., 2019; Heathers, 2014).

Moreover, the task force guidelines highlighted the interest of the representation of LF and HF metrics in normalized units, which was explained by the fact that this emphasized the controlled and balanced behavior of the two branches of the ANS. They also underscored its ability to alleviate the effect of changes in total power of the LF and HF component values (Heart rate variability: standards of measurement, physiological interpretation and clinical use. Task Force of the European Society of Cardiology and the North American Society of Pacing and Electrophysiology (1996)). However, the task force highly recommended that normalized units should always be quoted with the corresponding absolute values to completely describe the distribution of power in the spectral components (Burr, 2007). This recommendation may have raised some confusion, while stating that normalized units are more akin to reflect the ANS activity that may absolute values do, but at the same time, they claimed the necessity to report absolute values besides their normalized units’ counterparts, without discussing the underpinning of this recommendation (Burr, 2007).

While the normalization process seems methodologically interesting, it is important to point out the paradox associated with the normalized spectral HRV values, which affects the physiological interpretations related to the ANS function. In fact, the indices LF (nu), HF (nu) are algebraically dependent and linearly associated, as the mathematical relationship can be exposed by their sum: (LF (nu) + HF (nu)) = (LF/(LF + HF)) + (HF/(LF + HF)) = (LF + HF)/(LF + HF) = 1. Therefore, each of the indices is predictable from the other: LF (nu) = 1 – HF (nu), and HF (nu) = 1 – LF (nu) (Burr, 2007). According to this linear relationship, it becomes clear that reporting both values is considered as redundant. In other words, reporting both values do not provide any additional information as the change in one is identical to change in the other (Burr, 2007).

For instance, if the value of LF (nu) is esteemed to be 20%, hence, the value of HF (nu) must be 80%. Nonetheless, this model is an oversimplification of the complex interplay between the sympathetic and parasympathetic divisions of the ANS. Therefore, physiologically referring to HF (nu) and LF (nu) as separate concepts is incorrect. Instead, the overall components resulting from the normalization process should be described as reflecting the same autonomic continuum (Burr, 2007; Heathers, 2014). Nevertheless, normalized spectral indices of HRV are often interpreted similarly to their absolute unit counterparts. Furthermore, it is important to recognize that the collinearity between the normalized indices (LF and HF) implies that the statistical significance of one may equivalently denote the statistical significance of the other (Burr, 2007; Heathers, 2014).

**FIGURE 1:**
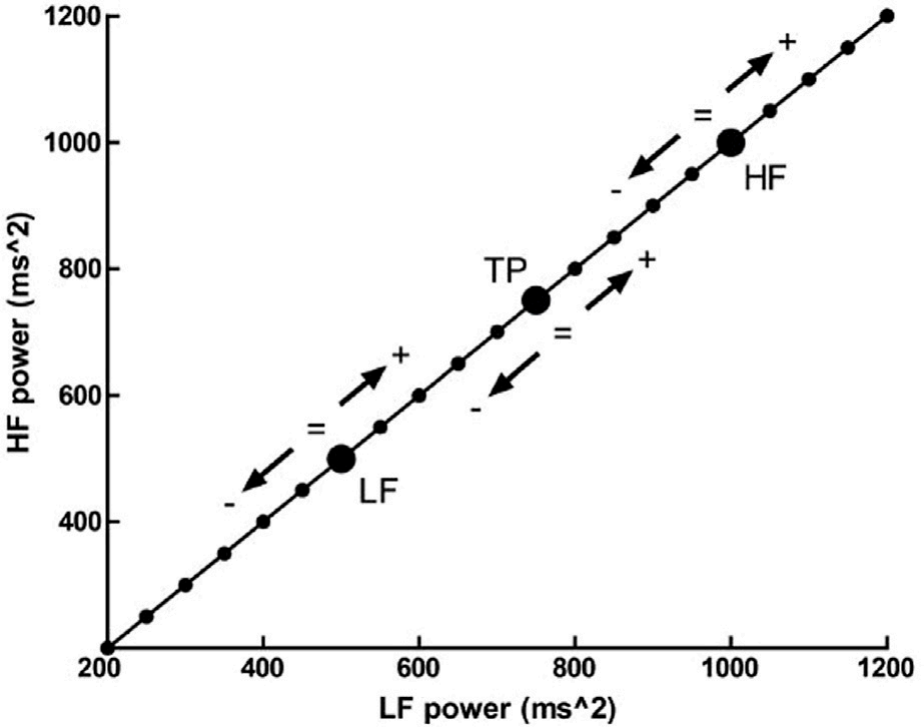
The outcome of a hypothetic experimental intervention. The participant has a LFnu = 0.33 (LF (ms^2^) = 500, and HF (ms^2^) = 1,000) which increases to LFnu = 0.5, which is defined by any point on the line of identity (i.e., LF (ms^2^) = HF (ms^2^)). Adapted from Heathers, 2014, licensed under CC BY 3.0.

Additionally, normalized spectral values’ use present an additional concern. Billman and colleagues (2013) have suggested, through a hypothetical subject’s values, that various patterns of change in individual spectral bands may lead to identical changes in proportion. A baseline value of LF (nu) = 0.33 increases to LF (nu) = 0.5 after an experimental intervention. This change in normalized units indicates not one possible change, but a continuum of potential changes that encompass an increase, decrease or no change in either total power, absolute LF or absolute HF power. Any point on the line of identity depicted in Figure 1 fulfills the condition of LF (nu) being equal to 0.5. However, the individual points represent distinct outcomes (Billman, 2013; Heathers, 2014).

Given the aforementioned reasons, it is clear that the reporting of normalized values, although their methodological interest, can significantly obscure the understanding of the ANS activity. Although this evidence-based assumptions, in numerous HRV studies, the presentation of the LF and HF in normalized values, without their absolute equivalents is a common practice (Chu et al., 2015; Kanegusuku et al., 2015). Providing both normalized and absolute values is crucial for a more accurate and comprehensive understanding of autonomic responses. Absolute values of spectral components provide essential insights into the magnitude of autonomic activity, which normalized values alone cannot supply. They help to differentiate between physiological changes due to actual modulation of autonomic activity and those resulting from changes in total power (Burr, 2007; Heart rate variability: standards of measurement, physiological interpretation and clinical use. Task Force of the European Society of Cardiology and the North American Society of Pacing and Electrophysiology (1996)). For instance, the reliance on normalized units might lead to the misleading conclusion that there is a significant change in autonomic balance wen, in fact, fluctuations could be due to changes in overall HRV signal power. Therefore, the combined use of normalized indices and their absolute counterparts is recommended to ensure accuracy, reduce redundancy, and enhance the reliability of interpretations in HRV studies.

Importantly, in some meta-analyses, relying on pooling data from multiple studies, normalized and absolute values are combined within the same analysis (Manresa-Rocamora et al., 2021). We believe that this practice may lead to statistical inconsistencies. Normalized and absolute measures are inherently different in their scales and interpretations, which can skew the combined effect sizes and introduce biases. From a physiological perspective, normalized units and absolute values represent different aspects of autonomic regulation. For example, the normalized LF/HF index provides the ratio of sympathetic to parasympathetic activity, independent of overall HRV magnitude. In contrast, the absolute values indicate the actual power within specific frequency bands, reflecting the total autonomic output. Therefore, when conducting meta-analyses on HRV measures, it is crucial to consider the aforementioned methodological concerns by conducting separate meta-analyses for normalized and absolute values.

Of note, in a previous meta-analysis on the effects of exercise training on heart rate variability (Amekran et al., 2024), separate analyses were conducted on frequency-domain HRV indices (absolute values and normalized values were analyzed separately). The result of the HF in absolute values was significant, whereas the result for the same parameter in normalized units was not significant. Moreover, none of the LF (nu), HF (nu), and LF/HF ratio results were significant, confirming the significance dependence between the indices.

## Conclusion

In summary, while HRV analysis is a method easily accessed to measure the ANS activity, the interpretability of its related metrics, especially in the frequency-domain, are still not fully understood, and present some caveats that could confound the mathematical and physiological interpretations, which may limit the capability of the method to draw inference into the autonomic function. Therefore, future research should carefully consider these concerns, so that they can lead to more accurate conclusions. Moreover, future HRV guidelines should appraise clear standards for the use of normalized and absolute values of HRV measures.
